# Development of a simple and valid nutrition screening tool for pediatric hospitalized patients with acute illness

**DOI:** 10.12688/f1000research.51186.1

**Published:** 2021-03-03

**Authors:** Hoda Atef Abdelsattar Ibrahim, Rasha Abdel-Raouf, Ahmed S. Zeid, Eman H. Elsebaie, Shaimaa Abdalaleem, Aya A. Amin, Hanna Aboulghar

**Affiliations:** 1 Pediatrics Department, Faculty of Medicine, Cairo University, Cairo, Egypt; 2 Public Health and Community Medicine, Faculty of Medicine, Cairo University, Cairo, Egypt; 3 Cancer Epidemiology and Biostatistics, National Cancer Institute, Cairo University, Cairo, Egypt

**Keywords:** Nutritional screening tools, appetite loss, acute illness, SGA, children

## Abstract

**Background: **Nutritional screening, intervention and assessment in patients with undernutrition are key components of any nutritional care. The goal of any nutritional assessment is to determine the specific nutritional risk(s). Presently, there are no guidelines on any ideal screening tool to be used on admission for identification of children that are at risk of developing malnutrition during their hospital stay. The objective of the study was to develop a valid and simple nutritional screening tool which can be used on hospital admission to identify pediatric patients at risk of malnutrition *.*

**Methods:** This study was cross sectional analytical that enrolled children (n:161) admitted with acute illness to the general wards at Cairo University Children Hospitals (CUCH). The answers to the developed questionnaire were compared to the Subjective Global Assessment (SGA), those with high accuracy (≥80%) were used for validity with anthropometric measures.

**Results:** In the ‘less than two years of age’ group, the simple and valid nutritional screening tools were the following questions: (Is there a problem during breast-feeding?), (Is there scanty breast milk?), (Is there appetite loss?). The simple and valid nutritional screening tools during the ‘early childhood’ group were the following questions: (Is there appetite loss?), (Is there any skipping of meals?), (Are they watching TV, videotapes and/or playing computer games for more than two hours/day?). The simple and valid  nutritional screening tools during the ‘late childhood’ group were the following questions: (Is there appetite loss?), (Are they watching TV, videotapes and/or playing computer games for more than two hours/day?).
**
**

**Conclusion: **The simple and valid nutritional screening tools differ according to age groups. The one which is valid in all ages is the question about the appetite loss.

## Introduction

Nutrition is an essential factor in the development, growth, and functioning of any child. Good nutritional status provides energy and nutrients which are essential to maintain our life and promote social, physical, emotional, and cognitive development ^1^.

Childhood malnutrition is known to be a global health concern as it is complicated with poor development and growth, as well as reduced educational outcomes of children and can have negative implications on their adulthood ^2, 3^.

The incidence of the undernutrition among the inpatients is seeming to be more than that noticed among the community; malnourished children have a comorbid association, and are vulnerable to developing further medical complications. In addition, evidence indicates that the nutritional state of the admitted ill children deteriorates during the hospital stay. Besides, the absence of any known nutritional screening tool in these circumstances could result in an underestimation of that condition ^4, 5^. The nutritional screening tool tries to identify the patients that are at nutritional risk, including not only the children who are undernourished, but also children who may develop any form of undernutrition during their hospital stay, and, the children whose prognosis can be improved as a result of the nutrition intervention ^6^.

The Subjective Global Nutritional Assessment (SGNA) is a systematic nutritional assessment method which had been validated. It contains items of a physical examination and nutrition-focused medical history, and it is the closest nutrition tool that is available for assessing the nutritional state in pediatrics. Although it is a systematic nutritional evaluation and screening tool, it does not give a chance for a simple and rapid pediatric nutritional screening tool owing to the time that is required for its completion ^7^. The characteristics of a nutritional screening tool are (1) sensitivity, specificity, and validity; (2) easy to use, simple, and without any need for training; (3) cheap, noninvasive and quick ^8^.

## Methods

### Study design

A cross sectional analytical study. ***Study population and setting:*** The current study was conducted at Cairo University Children Hospitals (CUCHs) for all children admitted with acute illness in the general wards over a period of 18 months.

### Sample size and technique

A convenient sample of all children (n =161) who were admitted to CUCHs were recruited for this study.

***Inclusion criteria:* 1-**Children with acute illness, in the first two days of admission (to avoid the effect of hospitalization and possible weight loss, to prevent bias). **2- **Both male and female genders. **3-** Approval of the parent or legal guardian.

***Exclusion criteria*: 1-** Children who have gross physical disabilities, mental retardation, and chronic illnesses. e.g., sickle cell disease and cystic fibrosis. **2- **Patients admitted for more than two days.

### Data collection and study tools

A semi-structured questionnaire form was used while interviewing the patients. The answers were compared with Pediatric Subjective Global Assessment (PSGA) validated tool ^7^. Answers with accuracy more than 80% were validated with the anthropometric measures. It covered the following items in the questionnaire : Appetite, Weight loss, Food allergies, Morbidity evidenced by recurrent hospitalizations, Enforcement to eat or not, Family eating together or not, Vitamins and minerals supplementations and Maternal deprivation, with focus on certain points in each stage as:

- *Infancy:* breast-feeding, feeding on colostrum, any problems during breast-feeding, developmental history, adverse reactions to vaccines, and feeding disorders. N.B The criteria for successful breast feeding was checked to exclude those if there were actual breastfeeding problems e.g., scanty breast milk.
- *Early childhood (2–8 years):* feeding disorders, eating out, skipping meals, child abuse, school achievement, functional impairment, mealtime atmosphere and watching TV or video-tapes and/or playing computer games for more than two hours per day.
- *Late childhood and early adolescence (8–12 years):* skipping meals, child abuse, school achievement, functional impairment, mealtime atmosphere and watching TV, or video-tapes and/or playing computer games for more than two hours per day.

Subjective global assessment evaluates the nutritional state which is based on the components of the history (any change in the weight, any change in the dietary intake, any gastrointestinal symptoms which had continued for more than two weeks, any change in the functional ability) in addition to the examination (subcutaneous fat loss, any wasting of muscles, edema in sacral/ankle area, and presence of ascites). Results were categorized subjectively into a global evaluation, where children had been rated into ῾well-nourished category᾽ (SGA A); ῾moderately malnourished category᾽ (SGA B); or ῾severely malnourished category᾽ (SGA C). The SGA is known to be the “gold standard” in comparison with the nutritional screening questions, for assessment of any malnutrition. The nutritional screening questions which had the highest accuracy, positive predictive and negative predictive values (≥80%) at predicting nutrition state (according to the comparison with SGA) were considered as the final nutrition screening tool. The Predictive Validity of our nutritional screening tool was established through comparison of the nutritional screening tool with anthropometric measures. When the tool is confirmed to be valid, it can be considered as a screening tool of the nutritional status.

### Definitions of malnutrition used in the study

- *Underweight:* when the weight for age is less than the mean by two standard deviations (SD) of the World Health Organization (WHO) Child Standards for growth or less than 5 ^th^ centile for age.
- *Stunting:* when the height for age is less than the mean by two standard deviations of the WHO Child Standards for growth or less than the 5 ^th^ centile for age.
- *Wasting:* when the weight for the height age is less than the mean by two standard deviations of the WHO Child Standards for growth or Body Mass Index (BMI) is less than the 5 ^th^ centile for age.
- *Overweight*: when the weight for the height is more than the mean by two standard deviations of the WHO Child Standards for growth or Body Mass Index( BMI )is more than 85 ^th^ centile for age
- *Obesity:* when the weight for the height is more than the mean by three standard deviations of the WHO Child Standards for growth or BMI is more than 95 ^th^ centile for age.

### Efforts to address and avoid potential sources of bias

1. The study was done on the admitted children in the first two days to avoid the effect of the hospital admission due to the possible weight loss owing to the possible appetite loss.

2. We excluded children with chronic illness as they may have external factors for malnutrition due to chronic disease, so we enrolled only patients with acute illnesses such as bronchiolitis, or glomerulonephritis as these diseases are acute and haven’t yet affected the nutritional state.

### Data analysis

After the step of data collection, the questionnaires with their answers were revised for the completeness and the logical consistency. The pre-coded data was subsequently entered on the computer using the Program of the Microsoft Office Excel for Windows. Data were then double checked and transferred to The Statistical Package of the Social Science, Version 21 (SPSS-V21). The provided graphs were consequently constructed using SPSS Program. All the statistical analysis was done using the two tailed tests and the alpha error of 0.05. P value less than or equal to 0.05 was identified to be statistically significant. Simple descriptive statistics (arithmetic mean and standard deviation) are used for the summary of the quantitative data and frequencies are used for qualitative data. Bivariate relationship was identified and displayed in cross tabulations and a subsequent comparison of proportions was performed through the chi-square and Fisher’s exact tests.

### Ethical considerations

The study was revised and approved by the scientific research committee and ethics of Cairo University, Faculty of Medicine (ethical clearance number, Ι-071017) and the study was done in accordance with Cairo University's laws for human research. Written informed consent for participation and publication of the patient ^'s^ details was obtained from parent/guardians/relative of the patients. The study was done after the explanation of its importance and the objectives of the study to the participants. Only subjects who clearly agreed were enrolled and those who refused after the explanation were excluded. 

## Results

The present study was conducted in CUCHs on children admitted with acute illnesses in the general wards. The answers to the developed questionnaire were compared to SGA, those with high accuracy (≥80%) were used for validity with anthropometric measures. Table 1 shows the characteristics of the study participants.

**Table 1.** Characteristics of the study participants.

Age group	Mean age ± SD *	Male n (%) **	Female n (%) **	Total n (%) ***
Infancy (≤ 2 years) (months)	11.09 ± 7.02	40 (60.6)	26 (39.4)	66 (41.0)
Early childhood (2-≤8 years) (years)	4.1 ± 1.57	37 (56.1)	29 (43.9)	66 (41.0)
Late childhood (>8 –12 years) (years)	10.41 ± 1.57	13 (44.8)	16 (55.2)	29 (18.0)

* SD: Standard Deviation. ** The percentage is raw percentage. ***The percentage is column percentage

### ῾Less than two years age῾ group

There were (n = 66) patients in this group, 40 males,and 26 females. Comparison of the answers of the developed questionnaire to those of the SGA was done to detect the questions which have the highest sensitivity, specificity, positive predictive value (PPV), negative predictive value (NPV), and level of accuracy. Table 2 summarizes the comparison results. Table 3 illustrates the validity. Table 4 illustrates the tool.

**Table 2.** Comparison between the screening questions and the subjective global assessment in the less than 2 years group.

Question	Sensitivity	Specificity	PPV	NPV	Accuracy	P-value
**1.Problem during breast- feeding**	70.0%	87.0%	70%	87%	81.8%	<0.001
**1.a Scanty breast milk**	55%	93.5%	78.6%	82.7%	81.9%	<0.001
**1.b Delayed weaning**	15%	100%	100%	73%	74.2%	0.007
**1.c Retracted nipple**	0%	95.7%	0%	68.8%	66.7%	0.344
**1.d Sore nipple**	5%	100%	100%	70.8%	71.2%	0.126
**1. e Thumb suckling**	5%	100%	100%	70.8%	71.2%	0.126
**1. f Early cessation due to pregnancy**	0%	97.8%	0%	69.2%	68.2%	0.506
**Breast- feeding**	95 %	2.2%	29.7%	50%	30.3%	0.538
**Appetite loss**	50%	95.7%	83.3%	81.5%	81.9%	<0.001
**Enforcement to eat**	40%	93.5%	72.2%	78.2%	77.3%	0.001
**Eating together**	55%	41.3%	28.9%	67.9%	45.5%	0.780
**Weight loss measured**	35%	95.7%	77.8%	72.2%	77.3%	0.001
**Weight loss, not measured**	15%	95.7%	60%	72.1%	72.1%	0.133
**Significant weight loss**	30%	100%	100%	76.7%	78.8%	<0.001
**Food allergy**	15%	91.3%	42.9%	71.2%	68.1%	0.445
**Morbidity like hospitalization**	15%	97.3%	75%	72.6%	72.7%	0.045
**Feeding disorders**	10%	97.8%	66.7%	71.4%	71.2%	0.161
**Developmental history delay**	20%	97.8%	80%	73.8%	74.3%	0.012
**Any reactions to vaccines**	10%	87%	25%	69%	63.6%	0.728
**Vitamins and/or, mineral supplements**	20%	80.4%	30.8%	69.8%	62.2%	0.967
**Feeding on colostrum**	95%	2.2%	29.7%	50%	30.3%	0.538

**Table 3.** Validation with anthropometric measures of the questions which have the highest sensitivity, specificity and accuracy by the SGA.

Question	Sensitivity	Specificity	PPV	NPV	Accuracy	P-value
**Problem during breast feeding**	66.7%	86.7%	70%	84.8%	80.3%	<0.001
**Scanty breast milk**	93.3%	52.4%	78.6%	80.8%	80.3%	<0.001
**Appetite loss**	47.6%	95.7%	83.3%	79.6%	80.4%	<0.001

**Table 4.** Development of the simple and valid nutritional screening tool in the῾ less than two years age᾽ group.

**Is there a problem during breast- feeding?**
**Is there scanty breast milk?**
**Is there appetite loss?**

### ῾The Early Childhood᾽ Group

There were (n = 66) patients in this group, 37 male, and 29 female. The questionnaire was answered. Then the questions were compared with SGA to detect the questions which have the highest specificity, sensitivity, positive predictive value (PPV), negative predictive value (NPV), and level of accuracy. Table 5 summarizes the comparison results. Table 6 illustrates the validity. Table 7 illustrates the tool.

**Table 5.** Comparison between the screening questions and the subjective global assessment in the early childhood group.

Question	Sensitivity	Specificity	PPV	NPV	Accuracy	P-value
Appetite loss	88.6%	100.0%	100%	88.6%	94%	<0.001
Skipping meals	88.6%	90.3%	91.2%	87.5%	89.4%	<0.001
Watching TV, video-tapes and/or computer games for more than two hours/day	77.1%	90.3%	90%	77.8%	83.3%	<0.001
Child abuse	60.0%	77.4%	63.2%	75%	68.2%	0.002
Enforcement to eat	45.7%	96.8%	94.1%	61.2%	69.7%	<0.001
weight loss, not measured	31.4%	90.3%	78.6%	53.8%	59.1%	0.031
Eating together	82.9%	3.2%	49.2%	14.3%	45.4%	0.067
Maternal deprivation	2.9%	100.0%	100%	47.7%	48.5%	0.343
Feeding disorders	22.9%	96.8%	88.9%	52.6%	57.6%	0.020
Eating out	45.7%	71.0%	64%	53.7%	57.5%	0.163
School achievement	40.0%	38.7%	42.4%	36.4%	39.4%	0.084
Functional impairment	31.4%	83.9%	68.8%	52%	56.1%	0.148
Vitamin and/or mineral supplements	2.9%	90.3%	25%	45.2%	43.9%	0.246
Morbidity like hospitalization	20.0%	96.8%	87.5.7%	51.7%	56.1%	0.037
Food allergy	11.4%	80.6%	40%	44.6%	44%	0.370
Significant weight loss(based on measurement)	8.6%	100.0%	100%	49.2%	51.5%	0.095
Weight loss, measured	8.6%	100.0%	100%	49.2%	51.5%	0.095
Fair mealtime atmosphere	25.7%	0.0%	0%	22.5%	13.6%	<0.001

**Table 6.** Validation with anthropometric measures of the questions which have highest sensitivity, specificity and accuracy with the SGA.

Question	Sensitivity	Specificity	PPV	NPV	Accuracy	P-value
Appetite loss	88.6%	100.0%	100.0%	88.6%	94.0%	<0.001
Skipping meals	88.6%	90.3%	91.2%	87.5%	89.4%	<0.001
Watching TV, video-tapes and/or computer games for more than two hours/day	77.1%	90.3%	90.0%	77.8%	83.3%	<0.001

**Table 7.** Development of the valid and simple nutritional screening tool during the early childhood group.

**Is there appetite loss?**
**Is there any skipping of meals?**
**Are they watching TV, video-tapes and/or** **computer games for more than two hours/day?**

### ῾The Late Childhood᾽ Group

There were (n = 29) patients in this group, 13 male and 16 female. The questionnaire was answered. Then the questions were compared with SGA to detect the questions which have the highest specificity, sensitivity, positive predictive value (PPV), negative predictive value (NPV), and level of accuracy. Table 8 summarizes the comparison results. Table 9 illustrates the validity. Table 10 illustrates the tool. 

**Table 8.** Comparison between the screening questions and the subjective global assessment in the late childhood group.

Question	Sensitivity	Specificity	PPV	NPV	Accuracy	P-value
**Appetite loss**	75.0%	92.3%	92.3%	75.0%	82.8%	<0.001
**Watching TV, video-tapes and/or computer** **games for more than two hours/day**	93.8%	69.2%	78.9%	90.0%	82.7%	<0.001
**Weight loss, measured**	25.0%	92.3%	80.0%	50.0%	55.2%	0.220
**Weight loss, not measured**	18.8%	100.0%	100.0%	50.0%	55.1%	0.099
**Significant weight loss**	25.0%	100.0%	100.0%	52.0%	58.6%	0.052
**Morbidity like hospitalization**	6.3%	100.0%	100.0%	46.4%	48.2%	0.359
**Enforcement to eat**	56.3%	69.2%	69.2%	56.3%	62.0%	0.170
**Eating together**	75.0%	0.0%	48.0%	0.0%	41.4%	0.052
**Maternal deprivation**	6.3%	100.0%	100.0%	46.4%	48.2%	0.359
**Eating out**	37.5%	61.5%	54.5%	44.4%	48.3%	0.958
**Skipping meals**	43.8%	92.3%	87.5%	57.1%	65.5%	0.031
**Child abuse**	43.8%	76.9%	70.0%	52.6%	58.6%	0.244
**School achievement satisfaction**	75.0%	23.1%	54.5%	42.9%	51.7%	0.904
**Functional impairment**	25.0%	76.9%	57.1%	45.5%	48.3%	0.904
**Fair mealtime atmosphere**	25.0%	7.7%	25.0%	7.7%	17.2%	<0.001

**Table 9.** Validation with anthropometric measures of the questions which have highest sensitivity and specificity and accuracy with the SGA.

Question	Sensitivity	Specificity	PPV	NPV	Accuracy	P-value
Appetite loss	66.7%	90.9%	92.3%	62.5%	75.9%	0.002
Watching TV, video-tapes and /or computer games for more than two hours/day	94.4%	81.8%	89.5%	90.0%	89.6%	<0.001

**Table 10.** Development of the valid and simple nutritional screening tool during the late childhood group.

Is there appetite loss?
Are they watching TV, video-tapes and /or computer games more than two hours/day

The nutrition screening tools for all age groups are illustrated in Table 11. 

**Table 11.** The nutrition screening tool.

The nutrition screening tool	
Less than two years	Is there problem during breast feeding?
	Is there scanty breast milk?
	Is there appetite loss?
The early childhood	Is there appetite loss?
	Is there any skipping of meals?
	Are they watching TV, video-tapes and/or computer games for more than two hours/day?
The late childhood	Is there appetite loss?
	Are they watching TV, video-tapes and/or computer games for more than two hours?

## Discussion

The nutritional state of any child is a determinant of the body composition and the functional state. Deficient states badly affect the patient's outcomes, mortality, morbidity, hospital stay, and re-admission rates. Thus, screening for risk factors which are known to be present with the deficiencies should be part of the evaluation of any child on admission. ^9, 10^.

The hospitalized children have high risks to develop severe malnutrition. The nutritional risk screening is an essential tool to maintain the nutritional status in any hospitalized patient owing to different reasons. For example, the energy need is increased and the subsequent decreased appetite is problematic. ^11^

It is important to know the inpatients children who are with nutrition risks so that the appropriate timely nutritional intervention and the planned treatment can be performed and the nutritional deterioration prevented to improve the health outcomes ^12^.

Hartman *et al.* recommended that “a valid and a simple nutritional screening tool appeared highly needed to improve the early cost effective identification of pediatrics who will get benefits later from the nutrition intervention” ^13^.

There are many nutritional screening tools which are currently being used in hospitals and the community, but most of them are difficult and sophisticated ^8^. In this work, we aimed to develop an ideal nutritional pediatric screening tool which can be easily and rapidly implemented, and with a high level of sensitivity ,specificity and good accuracy in the identification of the nutritional risks together with the nutrition-related outcomes.

Unfortunately, there is no previous study that has used the Pediatric Subjective Global Assessment as a gold standard method to compare the screening tools on hospital admission of pediatric patients to detect the tools which have the highest sensitivity, specificity, and accuracy. There is a lot of debate among doctors and professionals on the way to validate the nutritional screening tools, especially if they have higher accuracy, which can predict the present nutritional status. Some hospitals have validated their nutritional screening tools using a full nutrition evaluation. Nevertheless, it is questionable if this is the standard tool, especially as not all countries have sufficient dieticians and their role might be different depending on the country ^14^. In this study, SGA was used as the gold standard to identify the nutritional screening tools with the highest accuracy in order to be validated with anthropometric measures. The Pediatric Subjective Global Assessment consists of both objective and subjective items in a physical examination and detailed questionnaire; then each child is categorized into (1) well-nourished category, (2) moderately malnourished category, or (3) severely malnourished category ^15^. 

Our study resulted in a simple, valid and effective nutritional pediatric screening tool for the early identification of at-risk admitted children who require thorough nutritional assessment and subsequent individualized nutritional intervention. In this study, we tried to detect the ability of the tool to pick up those children who have malnutrition.

 In all age groups, the screening tool question about lost appetite was one of the most sensitive, specific, and accurate questions in prediction of the nutritional risk.

Our present study is in line with a study done on a simple nutritional screening tool for hospitalized pediatric children after checking the accuracy of a new, rapid, and simple pediatric nutritional screening method. The question (Has child been feeding less during the last weeks?) was one of the most sensitive and specific questions in detection of the patients with nutrition risks. On the other hand, it disagrees with the rest of questions screening tool as they were (Has the child lost weight unintentionally?), and (Has the child had poor or unsatisfied weight gain during the last months?). This difference may be due to the accuracy in weight detection objectively by the mothers ^16^.

In this study, we found that one of the nutritional screening tools was about the appetite loss. There was a study that used STAMP (Screening Tool Assessment of Malnutrition in Pediatrics) for the determination of the malnutrition and malnutrition risks in the pediatric primary health care setting. One of the items of this screening tool was (what is the nutritional intake of the child) which may be related to his appetite. However, it incorporated weight and height (anthropometric measures) in this screening tool unlike the case in our study. That is because we aimed to find a quick screening tool. Thus, we didn’t consider the anthropometric measures in the tool ^17^.

This study agrees with a previous study that found that one of the nutritional screening tools among the adult hospitalized patients was (Have you had poor eating because of the presence of a decreased appetite?), but disagrees with that the other tools were (Have you lost weight unintentionally?), (If yes, how much weight have you actually lost?). This difference may be due to the age group difference ^18^.

This study is very near to a previous study that was worked on the evaluation of the screening tool for further assessment of malnutrition in children. Significant predictors of the nutritional risks were decreased dietary intakes which was due to a recent poor appetite. Other predictors found were reported ^19^. 

This study agrees with a study that was done to detect the nutritional screening tool. One of the main principles of nutritional screening was about reduced intake due to a poor appetite. It disagrees with it in that the other principle was about the severity of the disease whether acute or chronic. This disagreement may be due to that in our present study we intentionally removed the effect of the chronic element on the disease which may affect the nutritional status. That is why we enrolled admitted patients with acute illnesses only ^20^.

In the ῾less than two years age᾽ group, it was found that the presence of any problems during breast- feeding or scanty breast milk were predictors of nutritional risks. They affect the nutritional status as seen in the underweight and the stunted groups. There was a significant association (P-value <0.001) between the presence of any problems or scanty breast milk and the prediction of any nutritional risk.

This study agrees with a study in which a relationship was found between the breast- feeding practices of mothers in Nairobi, and the nutritional state of their children aged between 0 to 24 months. There was a significant and noticeable association between the continuation of breast-feeding for a child less than 24 months and those underweight and stunting. Children who had stopped breast-feeding were more than three or four times likely to be underweight in comparison with those who continue breastfeeding ^21^.

This study is closely located to a previous study that was done in a rural area, under administrative control of the tertiary care hospital. All lactating mothers who have babies up to one year were participants in the study. They found a negative relationship between exclusive breast feeding and the prevalence of underweight and stunted children. Their conclusion was based on assumption that there is no breastfeeding problems in nourished children receiving exclusive breast feeding ^22^.

The current study found that, in the early childhood group, one of the predictors of nutritional risks was the question about skipping meals

This study agrees with a previous one which found that more subjects of the non- breakfast skippers were found to have BMI in the normal range than breakfast skippers. Unlike our study, they found that more of the breakfast skippers were overweight than the non-breakfast skippers. This may be a result of more consumption of junk food, i.e., high in saturated fat, by the breakfast skippers ^23^.

This study is similar to a previous one which found that there is an association between meal skipping and malnutrition. Among the skippers, 12.1% of children were in the underweight category and 10.3% of the children were overweight. The malnutrition is represented in the underweight and the overweight groups ^24^.

In the present study it was found that in the early and late childhood groups, there is a significant association between watching TV, video-tapes, and/or playing computer games for more than two hours per day and the presence of a nutritional risk with (P -value <0.001).

This agrees with a study which found that the older age of the child, the presence of multiple televisions at home, having her or his own television ,and number of hours spent inside watching television at the weekend were essentially associated with increased risk of childhood obesity ^25^.

The current study also agrees with a previous study that was done on an Egyptian cross-sectional survey on children aged between 6 and 17 years in Manshit El Gamal region of Fayoum Governorate. Increasing age and not eating breakfast were associated risks for stunting, whereas the incidence of obesity was higher in the children who eat while watching TV ^26^.

In the present study, no significant association was found between history of weight loss and the nutritional status of the child.

This disagrees with a previous study in that they aimed to detect a score to be used on hospital admission to pick up patients who are at risk of acute malnutrition during the hospital stay ^27^. The nutritional risk was evaluated within 48 hours of admission. The nutritional predictor score consisted of history of weight loss, decreased food intake, disease severity divided into Grades 1, 2 and 3 and pain. This disagreement may be owed to the fact that the present study didn’t use a scoring system in the questionnaire.

### Limitations

Some limitations to our study should be mentioned which, may in turn lead to the limitation of its generalizability to the total population. For example, the study sample was limited to Cairo University Children Hospitals; a broader geographic sample may lead to different results. Furthermore, this study has used a cross sectional design, thus a rather prospective one can help in making other predictions through follow up. One limitation of the Subjective Global Assessment to be mentioned is the limitations of attempts to only categorize undernutrition. Obese patients are effectively categorized as normal.

## Conclusion

This study has established the validity of a simple nutrition screening tool that can be implemented on infants and children at the time of admission to hospital. In the ῾less than two years of age᾽ group: the simple and valid nutritional screening tools were: (Is there a problem during breast- feeding?), (Is there scanty breast milk ?), (Is there appetite loss?).In ῾the early childhood group᾽: the simple and valid nutritional screening tools were: (Is there appetite loss?), (Is there any skipping of meals?), (Are they watching TV, video-tapes and/or playing computer games for more than two hours/day). The ῾late childhood᾽ group: the simple and valid nutritional screening tools were: (Is there appetite loss?), (Are they watching TV, video-tapes and/or playing computer games for more than two hours/day).

## Data availability

### Underlying data

Open Science Framework: Development of a simple and valid nutrition screening tool for pediatric hospitalized patients with acute illness, https://doi.org/10.17605/OSF.IO/6YTMN ^28^

Data are available under the terms of the Creative Commons Zero "No rights reserved" data waiver (CC0 1.0 Public domain dedication).
