# Supplementary material for: Development of a simple and valid nutrition screening tool for pediatric hospitalized patients with acute illness
Source: F1000Res. 2021 Mar 3;10:173. [Version 1] doi: 10.12688/f1000research.51186.1 (PMC8080976; doi:10.12688/f1000research.51186.1)
Supplement: Peer review history

# Peer review: Reviewer response for version 1

**DOI:** 10.5256/f1000research.54320.r83394

**Published:** 2021-04-23

**Authors:** ‪Areej Alkhaldy

**Affiliations:** 1 Clinical Nutrition Department, Faculty of Applied Medical Sciences, King Abdulaziz University, Jeddah, Saudi Arabia

It has been a great honour to review the article entitled “Development of a simple and valid nutrition screening tool for pediatric hospitalized patients with acute illness.” The paper aims to develop a valid and simple nutritional screening tool which can be used on hospital admission to identify pediatric patients at risk of malnutrition. The paper is interesting; however, there are some comments that need to be addressed to improve it.

**Abstract: **

- The age, sex, and groups need to be indicated in the abstract.
- The results need to be improved and expanded along with numbers.
- The conclusion needs to be rewritten. What is the main message regarding the use of these screening tools?

**Introduction: **

- The introduction was written in a consistent manner, yet you need to add more information including recent research in the area of SGA and pediatrics. There is a need to focus more on the nutrition screening tool for pediatric patients than general ones for adults.
- At the end of the last paragraph, the objectives need to be expressed in a clear manner and include the rational of conducting the study.

**Methods: **

- You need to include more information in the sample size section. How was the sample size calculated in terms of population size, source of the data, and references of the equation or website, or program you used to calculate the sample size?
- Explain the validation process in more detail.
- Briefly explain the questionnaire (for example: sections and numbers of questions)  
- Define abbreviations at first mention; the abbreviation should always be used in the rest of the manuscript instead of the complete term. All group abbreviations need to be indicated and explained in the methods.

**Results: **

- Table 2, 5, and 8 could be combined and compared
- Footnotes need to be added to all tables (example PPV and NPV)

**Discussion:**

- You need to discuss your findings in the context of the other studies in the same area.
- The manuscript requires minor language editing throughout.

Is the work clearly and accurately presented and does it cite the current literature?

Yes

If applicable, is the statistical analysis and its interpretation appropriate?

I cannot comment. A qualified statistician is required.

Are all the source data underlying the results available to ensure full reproducibility?

Partly

Is the study design appropriate and is the work technically sound?

Yes

Are the conclusions drawn adequately supported by the results?

Yes

Are sufficient details of methods and analysis provided to allow replication by others?

Partly

Reviewer Expertise:

Clinical Nutrition

I confirm that I have read this submission and believe that I have an appropriate level of expertise to confirm that it is of an acceptable scientific standard.

# Peer review: Reviewer response for version 1

**DOI:** 10.5256/f1000research.54320.r83395

**Published:** 2021-04-22

**Authors:** Ali El-shafie

**Affiliations:** 1 Department of Pediatrics, Faculty of Medicine, Menoufia University, Shebin El-Koum, Egypt

Thank you for your kind invitation to be a reviewer for this study.

 The study investigated a nutritional screening tool for pediatrics. The researchers tried to reveal the sensitivity, specificity and validity of this tool. Although the results of the study are intriguing, few flaws remain in the paper.

 Title: It would be better that more specific term would have been chosen for the title of study. I suggest you describe the tool as it is rapid. In addition, I suggest you describe those pediatrics as having acute malnutrition as this way of description is more specified. Thus, the title would be (Development of a rapid and valid nutritional screening tool for pediatric hospitalized patients with acute malnutrition).

 Abstract: The first sentence would have been removed as the paper didn’t incorporate any methods for nutritional intervention. 

 Introduction: The subjective global assessment is needed to be further explained. In addition, the basis of choosing the questionnaire for each group and previous studies that used similar questionnaires would have been better introduced.

 Method: The logic for calculating the sample size is missed in this study. The calculation of sample size or a power analysis is better to be added. The acute illnesses would have been prescribed and correlations between them and types of malnutrition would have been addressed. Regarding the SGA, its method is better to be mentioned clearly. 

 Results: I noticed that there are no graphs to explain the groups of malnutrition revealed in the study. I think it was better to add these graphs. Concerning the applied questionnaire, what is meant by "weight loss measured", it would have been better explained. Furthermore, the question of "food allergy" is not clear as symptoms of food allergy that you asked should be associated with the questionnaire 

 Discussion: The first four paragraphs could be removed as they are repeated. The aim of the study should have been mentioned at the end of the Introduction part instead of at the beginning of the Discussion one.

Is the work clearly and accurately presented and does it cite the current literature?

Yes

If applicable, is the statistical analysis and its interpretation appropriate?

Partly

Are all the source data underlying the results available to ensure full reproducibility?

Yes

Is the study design appropriate and is the work technically sound?

Yes

Are the conclusions drawn adequately supported by the results?

Yes

Are sufficient details of methods and analysis provided to allow replication by others?

Partly

Reviewer Expertise:

NA

I confirm that I have read this submission and believe that I have an appropriate level of expertise to confirm that it is of an acceptable scientific standard.

# Peer review: Reviewer response for version 1

**DOI:** 10.5256/f1000research.54320.r80755

**Published:** 2021-03-22

**Authors:** Osama El-Asheer

**Affiliations:** 1 Paediatric Department, Assiut University, Assiut, Egypt

I have reviewed the document from my Egyptian colleagues with great interest. This paper is well written and addresses an important topic which is the development of a simple and valid nutrition screening tool in pediatric hospitalized patients with acute illness. The article is clear and of relevance also because there is a need for this important tool to improve the prognosis and outcomes of pediatric diseases. Furthermore, the tool respects the age groups and also respects the time as it is rapid (unlike the subjective global assessment and the anthropometric measures) and applicable.

 Although the reliability of this tool was not achieved, there are several important conclusions from this article. For example, appetite loss is the most consistent tool in all age groups. However, a scoring system for this appetite loss would have been done to improve the quality of this tool. Very important: watching television and computer games for more than 2 hours per day could represent a major nutritional risk for older children. Besides, respecting the meal time and the avoidance of skipping meals can greatly keep children away from malnutrition. Problems in breastfeeding can negatively affect the growth of infant and have earlier implications on the nutritional parameters. Correctly the authors exclude the chronic diseases to exclude bias as the chronic course could cause organic failure to thrive.

 To improve paper readability, I suggest the following:

- Abstract: Detailed questionnaire should be removed from Results
- Please clarify the method of getting the sample size.
- Please clarify why you did not compare with other screening tools like STAMP or STRONG Kids scoring in addition to SGNA
- Please clarify the base of age classification 2-8 and 8-12
- Results: tables are so much and should be summarized into 4-5 tables
- The discussion should be shorter and more focused to the point. It should be more aimed more than this. You can remove the first six paragraphs in the discussion as you mentioned similar ones in the introduction.
- I suggest also to update the references.  
- As a further question: Did the authors observe the nutritional lab assessment?

Is the work clearly and accurately presented and does it cite the current literature?

Yes

If applicable, is the statistical analysis and its interpretation appropriate?

I cannot comment. A qualified statistician is required.

Are all the source data underlying the results available to ensure full reproducibility?

Yes

Is the study design appropriate and is the work technically sound?

Yes

Are the conclusions drawn adequately supported by the results?

Yes

Are sufficient details of methods and analysis provided to allow replication by others?

Yes

Reviewer Expertise:

Professor Of Pediatrics - Assiut University & Head of clinical nutrition unite - Assiut University children hospital

I confirm that I have read this submission and believe that I have an appropriate level of expertise to confirm that it is of an acceptable scientific standard, however I have significant reservations, as outlined above.

# Response

**Published:** 2021-03-22

**Authors:** Hoda Atef

**Affiliations:** Cairo University, Egypt

**Dear Professor , DR :  Osama El-Asheer**

 Thank you for your valuable comments

1. Regarding the results : we added the detailed questionnaire to collectively gather the ways for the  aimed tool
2. Regarding the sample size : it depended on the time of study and all patients who fulfilled the inclusion criteria were enrolled in the study
3. Regarding the comparison with other tools like STAMP or STRONG Kids scoring, we didn’t use them as this wasn’t our aim of the  study . Our aim was to find  a simple and a valid tool . The process of validity required  high sensitivity and specificity which needed a comparison with any valid tool such as SGA
4. Regarding the base of age classification . WHO (World Health Organization ) defines the early childhood group from 0-8 years . I further categorized  the first two years as a separate group as they have different feeding (e.g. breast feeding ) and its problems are present during this period which is a distinctive period in the childhood
5.  Regarding the tables , our aim was to explain in details .However, they could be further shortened
6. Regarding the discussion ,we intended to introduce our work before comparing it with the similar articles in the discussion . However, it could be further shortened.
7. Regarding the references , we tried to update them till 2020
8. Regarding the lab comments , they weren’t observed as this wasn’t  the aim . The aim of the study was to develop a rapid and simple tool. The labs would  take time.

# Peer review: Reviewer response for version 1

**DOI:** 10.5256/f1000research.54320.r80762

**Published:** 2021-03-08

**Authors:** Andrew S Day

**Affiliations:** 1 Department of Paediatrics, University of Otago Christchurch, Christchurch, New Zealand

This MS describes the development of a short nutritional risk screening tool, with comparison to a larger tool

 Specific Comments:

1. The population is relatively small - larger numbers required to clearly demonstrate the utility of this tool. Especially with the three subgroups
2. There was no depiction of the current nutritional state, of the underlying medical issues, the length of stay or the change in weight during the stay. NRS are focused on identifying those at risk of nutritional compromise during admission - this doesn't include that focus
3. The METHODS should describe how the study was done. The number of subjects is a result and should be moved accordingly
4. There are a large number of tables - are these all required? some could be supplementary instead and some deleted
5. The DISCUSSION should be shortened and more focused. When using et al, the reference number must follow directly afterwards. Some sentences are awkward and could be improved with revision

Is the work clearly and accurately presented and does it cite the current literature?

Partly

If applicable, is the statistical analysis and its interpretation appropriate?

Yes

Are all the source data underlying the results available to ensure full reproducibility?

No source data required

Is the study design appropriate and is the work technically sound?

Partly

Are the conclusions drawn adequately supported by the results?

Yes

Are sufficient details of methods and analysis provided to allow replication by others?

Yes

Reviewer Expertise:

paediatric gastroenterology and nutrition

I confirm that I have read this submission and believe that I have an appropriate level of expertise to state that I do not consider it to be of an acceptable scientific standard, for reasons outlined above.

# Response

**Published:** 2021-03-19

**Authors:** Hoda Atef

**Affiliations:** Cairo University, Egypt

Dear : Andrew S Day

 Thank you for your valuable comments

 1.Regarding the point number 1: the sample size was dependent on the time of the study.

 In addition, we excluded patients with chronic illnesses to exclude bias as the chronic nature could cause malnutrition. These could be the causes of  a smaller sample size as the inclusion criteria were very precise

  Of course, further studies are needed to validate the tool and our study may be the point of the start for these further researches.

 2. Regarding the point number 2: There was a depiction for the current nutritional status . You can get it in the supplementary files. For example, there were underweight, wasted and short status groups. Regarding the length of hospital stay, it wasn’t our aim as the study aim is to find a tool on hospital admission. Further studies are required to apply it on the hospital length stay.

 3. Regarding the point number 3, please explain more, what do you mean?

 4. Regarding the point number 4, our aim was to explain all data but the tables could be further summarized 

 5. Regarding the point number 5, we tried to comment on the results as much as possible and compare them with the relevant articles. However, the discussion could be further shortened. 

 Thank you so much 

 Sincerely

 Hoda Atef
